# Supplementary material for: Large and tunable magnetoresistance in van der Waals ferromagnet/semiconductor junctions
Source: Nat Commun. 2023 Sep 4;14:5371. doi: 10.1038/s41467-023-41077-0 (PMC10477182; doi:10.1038/s41467-023-41077-0)
Supplement: Supplementary file 1 — Supplementary Information [file 41467_2023_41077_MOESM1_ESM.pdf]

Supplementary Information

Large and tunable magnetoresistance in van der Waals Ferromagnet/Semiconductor junctions

Wenkai Zhu[1,2,9], Yingmei Zhu[3,9], Tong Zhou[4], Xianpeng Zhang[5], Hailong Lin[1,2], Qirui Cui[3], Faguang Yan[1], Ziao Wang[1,2], Yongcheng Deng[1], Hongxin Yang[3]✉, Lixia Zhao[1,6]✉, Igor Žutić[4]✉, Kirill D. Belashchenko[7]✉, Kaiyou Wang[1,2,8]✉

[1]State Key Laboratory of Superlattices and Microstructures, Institute of Semiconductors, Chinese Academy of Sciences, Beijing 100083, China

[2]Center of Materials Science and Optoelectronics Engineering, University of Chinese Academy of Sciences, Beijing 100049, China

[3]National Laboratory of Solid State Microstructures, School of Physics, Collaborative Innovation Center of Advanced Microstructures, Nanjing University, Nanjing 210093, China

[4]Department of Physics, University at Buffalo, State University of New York, Buffalo 14260, United States

[5]Department of Physics, University of Basel, Basel CH-4056, Switzerland

[6]Tiangong University, Tianjin 300387, China

[7]Department of Physics and Astronomy, Nebraska Center for Materials and Nanoscience, University of Nebraska-Lincoln, Lincoln, Nebraska 68588, USA

[8]Beijing Academy of Quantum Information Sciences, Beijing 100193, China

[9]These authors contributed equally: Wenkai Zhu, Yingmei Zhu.

✉e-mail: hongxin.yang@nju.edu.cn; lxzhao@tiangong.edu.cn; zigor@buffalo.edu; belashchenko@unl.edu; kywang@semi.ac.cn

Table of Contents

Supplementary Note 1. Characterization of phase structure of GaSe

Supplementary Note 2. The thickness of the GaSe barrier layers

Supplementary Note 3. Tunneling mechanism of the MTJ devices

Supplementary Note 4. The measured TMR in the MTJ devices with different GaSe-layer thickness

Supplementary Note 5. The reproducibility of the barrier-thickness-dependent TMR

Supplementary Note 6. The bias-dependent TMR in the MTJ devices

Supplementary Note 7. The spin-resolved DOS simulated by DFT

Supplementary Note 8. The temperature-dependent TMR in the MTJ devices

References for Supplementary Information

Supplementary Note 1. Characterization of phase structure and conductivity of GaSe

Gallium selenide (GaSe) single crystals are a typical two-dimensional (2D) layered metal monochalcogenide with an indirect bandgap energy of ~2 eV in the bulk[1,2]. To characterize the crystal quality of GaSe, we measured the Raman and PL spectrum of the bulk GaSe. As shown in **Figure S1a**, the Raman spectrum of the bulk GaSe shows peaks at ~135 cm^{-1}, 214 cm^{-1}, 245 cm^{-1}, and 308 cm^{-1}, corresponding to the A_{1g}^2, E_{2g}^1, E_{1g}^2, and A_{1g}^2 vibration mode of GaSe, respectively. The PL measurements indicates the bandgap energy of the bulk GaSe is ~2 eV (**Figure S1b**). This notation is consistent with earlier works on bulk ε-GaSe[1,3].

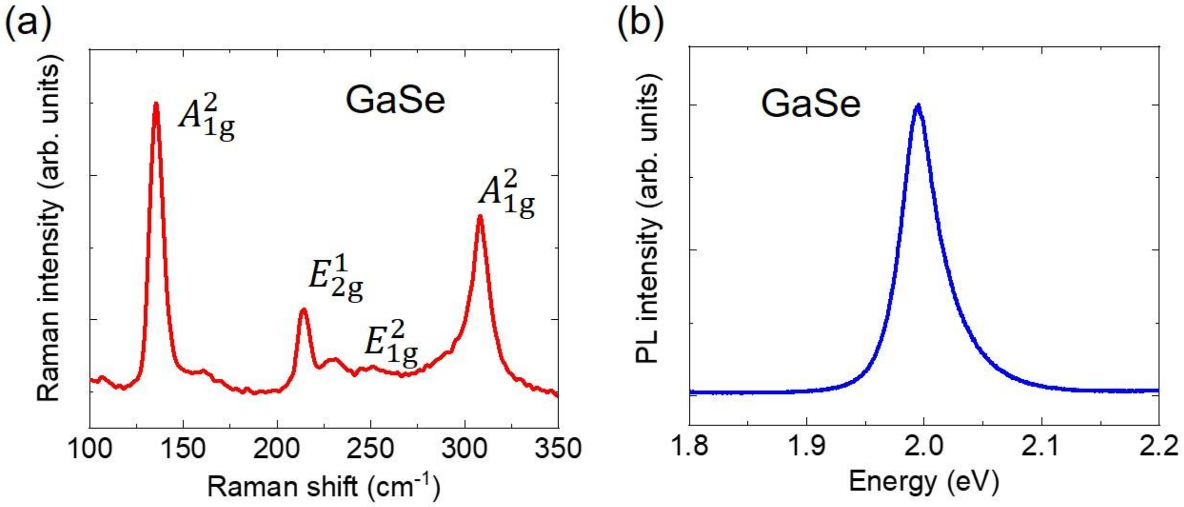

Figure S1. (a) The Raman spectrum and (b) Photoluminescence (PL) spectrum of a bulk GaSe flake (measured with 532 nm laser excitation at room temperature). The spectrum shows the band edge emission of GaSe, which is centered at about 2 eV.

Supplementary Note 2. The thickness of the GaSe barrier layers

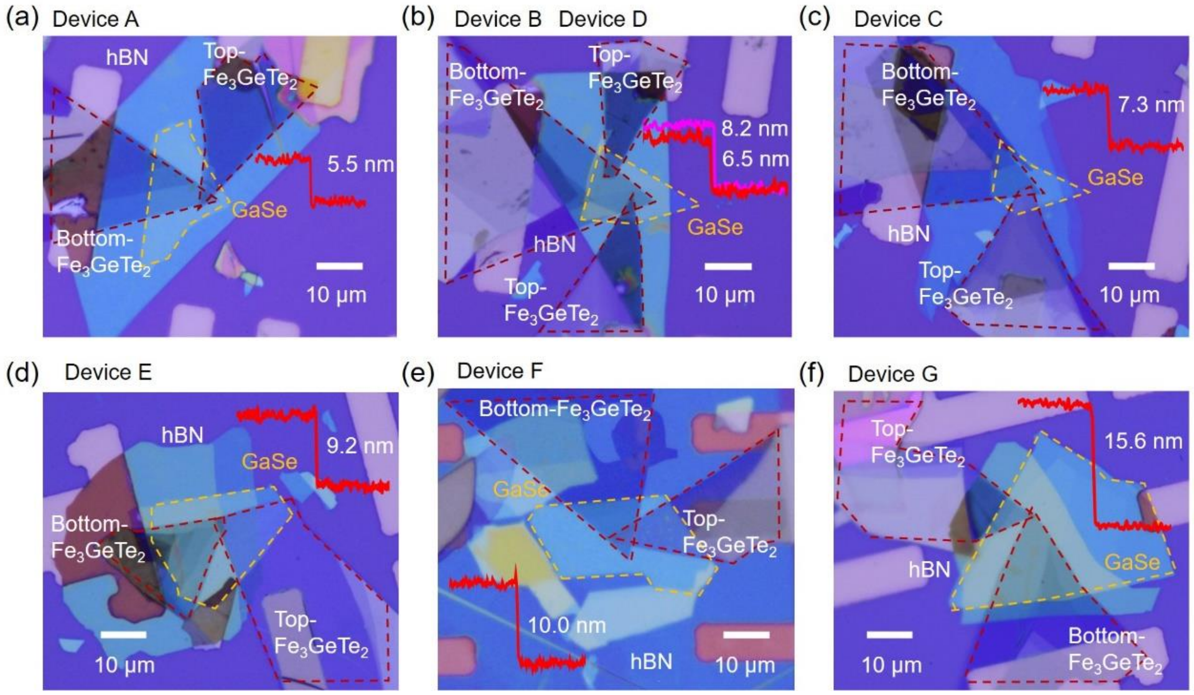

Figure S2. (a-f) The optical images of the devices A-G, respectively. The insets of the figures (a-f) show the scanning thickness of GaSe flakes for devices A-G by AFM measurements. The FGT and GaSe flakes are outlined with red and orange dotted lines respectively. The scale bar is 10 μm.

As shown in **Figure S2a-f**, the optical images of the devices A, B, C, D, E, F and G, where The FGT and GaSe flakes are outlined with red and orange dotted lines respectively. The insets show the scanned thickness of the GaSe flakes by atomic force microscope (AFM), indicating the devices A, B, C, D, E, F and G is about 5.5, 6.5, 7.3, 8.2, 9.2, 10.0 and 15.6 nm, respectively. Due to the thickness of monolayer of GaSe is about 0.93 nm[4], thus the devices A, B, C, D, E, F and G have 6-layers, 7-layers, 8-layers, 9-layers, 10-layers, 11-layers and 17-layers of GaSe monolayer, respectively.

Supplementary Note 3. Tunneling mechanism of the MTJ devices

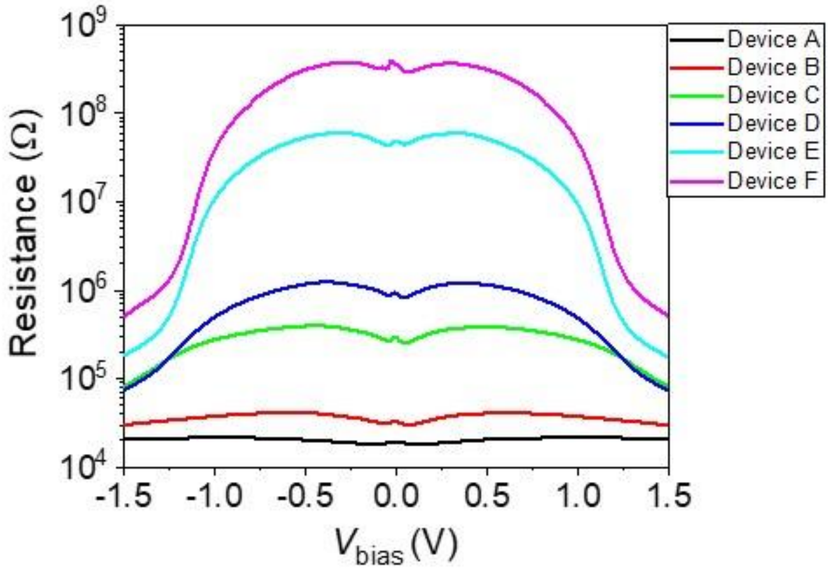

Figure S3. The resistances of the devices change with the bias voltage. In different devices, the change trend of the resistance with the bias voltage is almost the same. Under a large bias voltage, the resistances of the devices show parabolic behavior, indicating that they are all tunneling devices.

We next analyze the tunneling mechanisms in the different devices. When the applied bias voltage is far less than the effective tunnel-barrier height ($V_{\text{bias}} \ll \phi_B$), the conduction mechanism is only direct tunneling, and current density-voltage dependence of direct tunneling is described by the Simmons' formula[5], that is,

$$J = \frac{3q^2(2m^*q\phi_B)^{1/2}V_{bias}}{2h^2d} \exp\left[-\frac{4\pi d(2m^*q\phi_B)^{1/2}}{h}\right], (1)$$

where q is the elementary charge, d is the barrier width, m^* is the effective electron mass (~ 0.1 m_0 in GaSe[6]), ϕ_B is the effective tunnel-barrier height and h is Planck's constant. When the applied bias voltage exceeds the effective tunnel-barrier height ($V_{\text{bias}} > \phi_B$), the tunneling mechanism is described by F-N tunneling[7-10], and the F-N tunneling current density can be expressed by the formula of

$$J = \frac{q^3V_{bias}^2}{8\pi hq\phi_B d^2} \exp\left[\frac{-8\pi d(2qm^*)^{1/2}}{3hV_{bias}}{\phi_B}^{3/2}\right]. (2)$$

To obtain the effective tunnel-barrier height information under different tunneling mechanisms, the above two formulas are linearized with the logarithmic scale, they can be written as:

$$ln\frac{J}{V_{bias}^2} = ln\left(\frac{3q^2(2m^*q\phi_B)^{\frac{1}{2}}}{2h^2d}\right) + ln\,(\frac{1}{V_{bias}}) - \frac{4\pi d(2m^*q\phi_B)^{1/2}}{h}$$ for direct tunneling. (3)

$$ln\frac{J}{V_{bias}^2} = ln\frac{q^3}{8\pi hq\phi_B d^2} - \frac{8\pi d(2qm^*{\phi_B}^3)^{\frac{1}{2}}}{3h}(\frac{1}{V_{bias}})$$ for F-N tunneling. (4)

Extracting from the J-V_{bias} data measured at 10 K (Figure 1b), the ln(J/V_{bias}^2) versus 1/V_{bias} curves for the different devices are shown in the **Figures S4a-f**, in which the red dotted line is fitted by the direct tunneling formula. Both device A and device B are dominated by direct tunneling in the range of 0-1.5 V bias. The devices C-F are dominated by direct tunneling under a small bias voltage, and gradually show F-N tunneling with the increase of bias voltage. Under a small bias voltage, the effective tunnel-barrier heights of the devices are obtained by fitting the Simmons' formula, which decreases slowly with the increase of the barrier thickness, and the effective tunnel-barrier height is ~0.9 eV (**Figure S4h**).

With the increase of the barrier thickness, the F-N tunneling become more and more obvious. For F-N tunneling, a plot of ln(J/V_{bias}^2) versus 1/V_{bias} should be linear. In addition, the slope of the F-N tunneling plots can be expressed as a function of the effective mass and the effective tunnel-barrier height:

$$\mathrm{slope} = -6.83 \times 10^9 \times d\sqrt{\left(\frac{m^*}{m_0}\right){\phi_B}^3}. \ (5)$$

As shown in **Figure S4g**, the F-N tunneling plots of ln(J/V_{bias}^2) vs 1/V_{bias} with narrow abscissa value for the different devices, where the red dotted line is the curve fitted by the F-N tunneling formula. Therefore, the effective tunnel-barrier height of devices C, D, E, F and G can be estimated as 0.42, 0.45, 0.90, 0.88 and 0.86 eV, respectively. The effective tunnel-barrier height is also ~0.9 eV except for devices C and D, indicating transport mechanism is related to the barrier thickness[8]. In devices C and D, the direct tunneling current is large and cannot be ignored due to the their relatively small barrier thickness. Under a large bias voltage, device C and device D are jointly affected by direct tunneling and F-N tunneling. Thus, using the F-N tunneling formula to fit the effective tunnel-barrier heights of device C and device D will deviate. Since the bandgap of bulk GaSe is ~2 eV, the energy band diagram of the device under direct and F-N tunneling mechanisms are shown in the inset of **Figure S4h**. The band alignment with lager tunnel-barrier between the ferromagnetic metal and semiconductor is crucial for improving the TMR ratio of the devices; otherwise, the spin polarization would be diminished dramatically due to the conductivity mismatch[11,12].

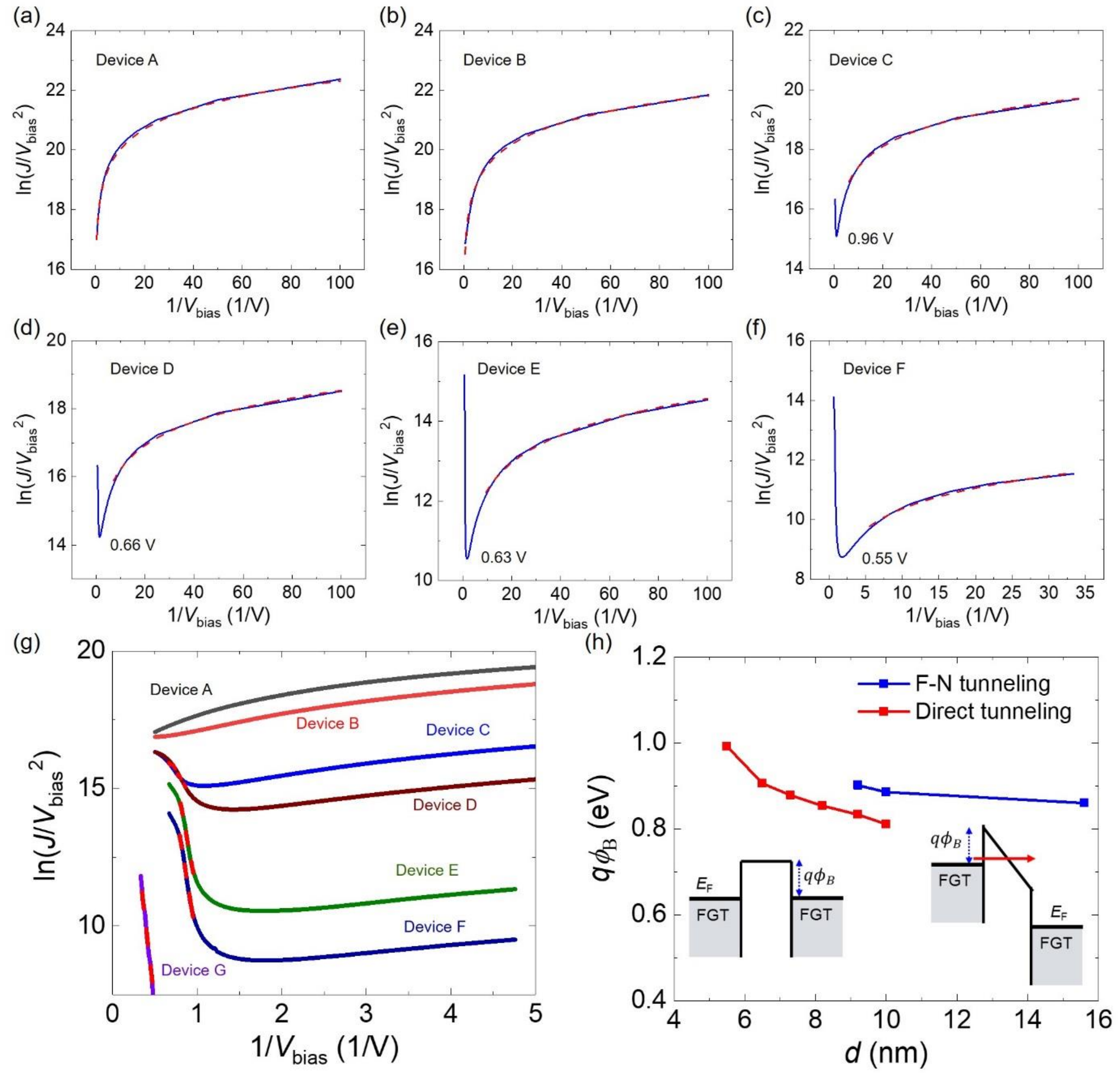

Figure S4. (a-f) The $\ln(J/V_{bias}^2)$ vs $1/V_{bias}$ curves for the different devices (blue line), and the red dotted line is fitted by the direct tunneling formula. (g) The F-N tunneling plots of $\ln(J/V_{bias}^2)$ vs $1/V_{bias}$ with narrow abscissa value for the different devices, where the red dotted line is the curve fitted by the F-N tunneling formula. (h) The effective tunnel-barrier height is extracted by fitting the direct tunneling formula and F-N tunneling formula respectively. The insets show the schematic diagram of direct tunneling under a small bias voltage and F-N tunneling mechanism under a large-bias voltage. The temperature is fixed at $T = 10$ K.

Supplementary Note 4. The measured TMR in the MTJ devices with different GaSe-layer thickness

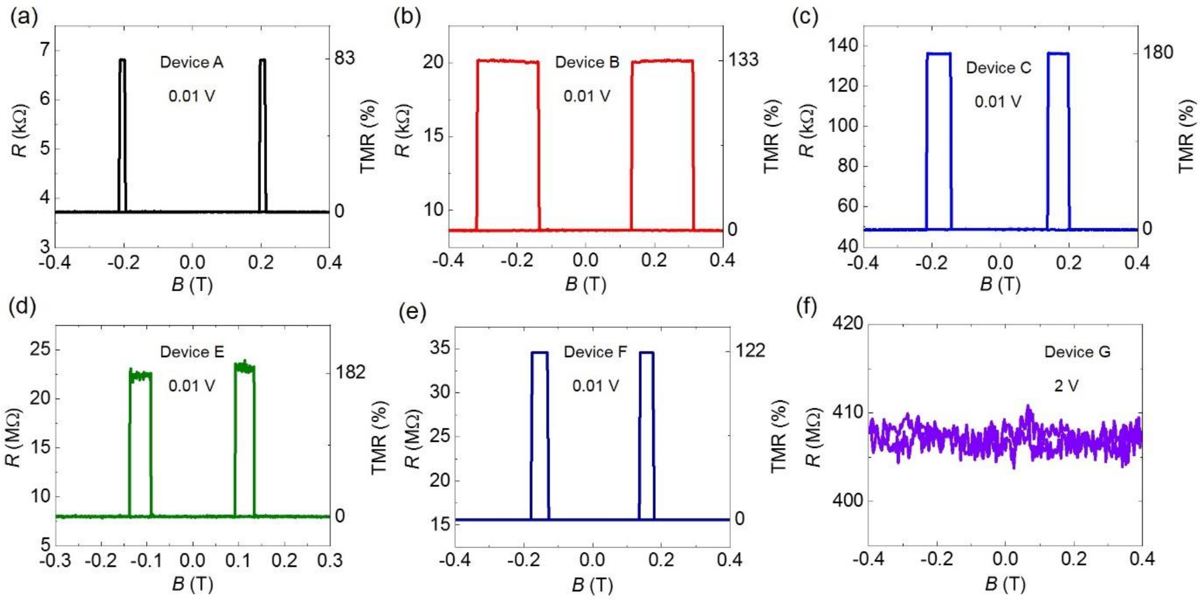

Figure S5. (a) *R*-*B* curves of device A at 10 mV. The R_P and R_{AP} of device A are 3.72 kΩ and 6.81 kΩ, with TMR ~83.1%. (b) *R*-*B* curves of device B at 10 mV. The R_P and R_{AP} of device B are 8.63 kΩ and 20.15 kΩ, with TMR ~133.5%. (c) *R*-*B* curves of device C at 10 mV. The R_P and R_{AP} of device C are 48.58 kΩ and 136.11 kΩ, with TMR ~180.2%. (d) *R*-*B* curves of device E at 10 mV. The R_P and R_{AP} of device E are 8.03 MΩ and 22.67 MΩ, with TMR ~182.3%. (e) *R*-*B* curves of device F at 10 mV. The R_P and R_{AP} of device F are 15.59 MΩ and 34.57 MΩ, with TMR ~121.7%. (f) *R*-*B* curves of device G under applied bias of 2 V. There is no TMR effect in device G. The temperature is fixed at 10 K.

Supplementary Note 5. The reproducibility of the barrier-thickness-dependent TMR

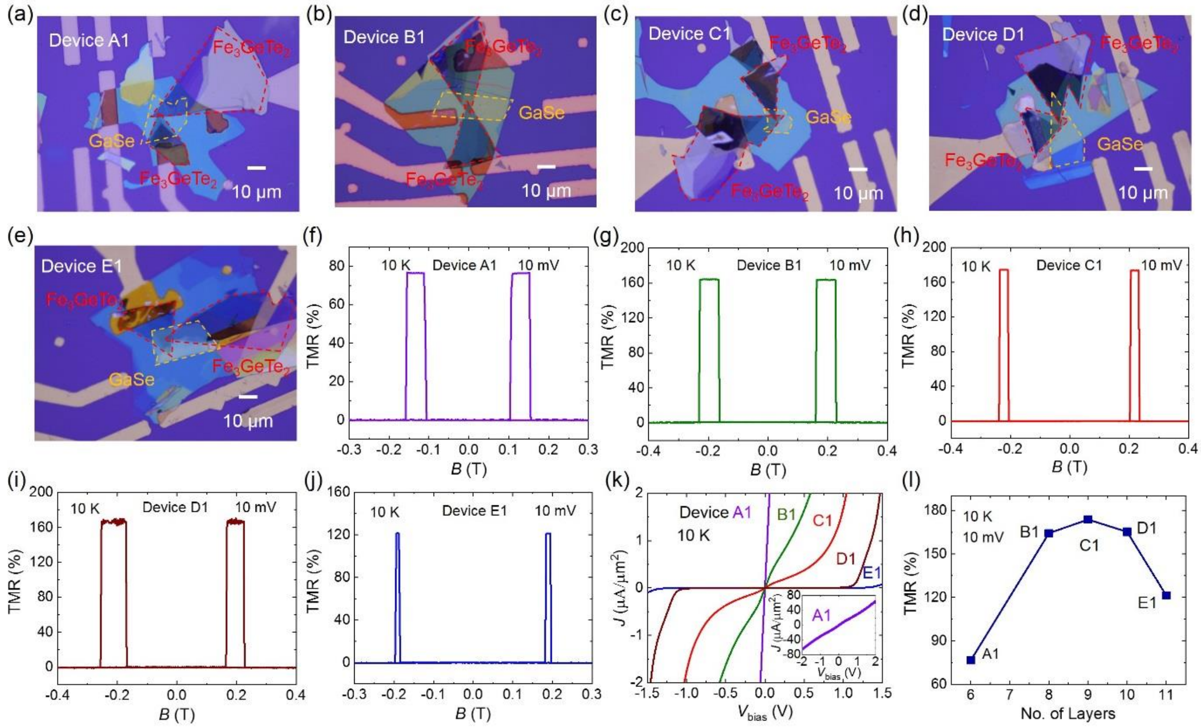

Figure S6 (a-e) The optical images of devices A1, B1, C1, D1, and E1, respectively. Scale bar: 10 μm. (f-j) The TMR-*B* curves of the devices measured at 10 K and 10 mV bias. (k) The J-V_{bias} curves at parallel state for the different devices. The inset shows the J-V_{bias} curve of the device A in a large bias range. (l) The extracted TMR value for the devices. The temperature is fixed at 10 K.

In order to confirm the behavior that TMR has a maximum value with varying the barrier layer thickness, we prepared another group of MTJ devices with different GaSe thickness ranging from 6-layers to 11-layers. Before preparing the ferromagnet/semiconductor heterojunction, we first determine the thickness of the used GaSe flakes through its optical contrast and AMF test results[13]. The optical images of devices A1, B1, C1, D1, and E1 are shown in **Figures S6a-e** respectively. The TMR-*B* curves of the devices measured at 10 K and 10 mV bias are shown in **Figures S6f-j**, respectively. The extracted TMR value is shown in **Figures S6l**. With the increased GaSe thickness, the TMR value first increases, then decreases, and the maximum value appears at 9-layer GaSe. As shown in **Figures S6k**, the J-V_{bias} curves at parallel state for different devices is still nonlinear and dispersed, indicting the transport is determined by the tunneling mechanism. In summary, the reproducibility of the barrier-thickness-dependent TMR characteristics has been proved.

Supplementary Note 6. The bias-dependent TMR in the MTJ devices

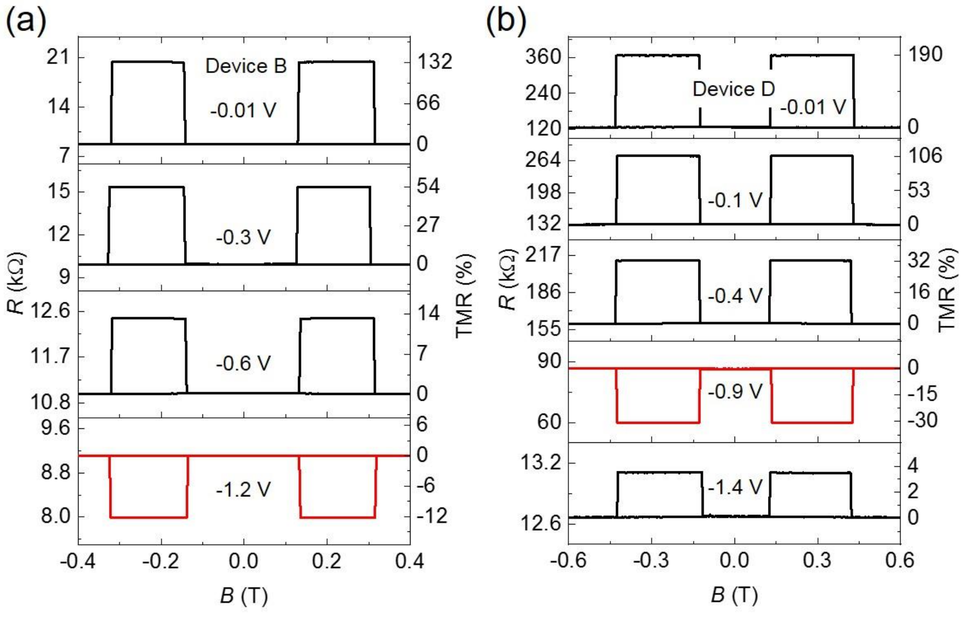

Figure S7. *R*-*B* curves and the corresponding TMR at various negative bias for devices B with 6.5-nm-thick GaSe (a) and D with 8.2-nm-thick GaSe (b) at 10 K.

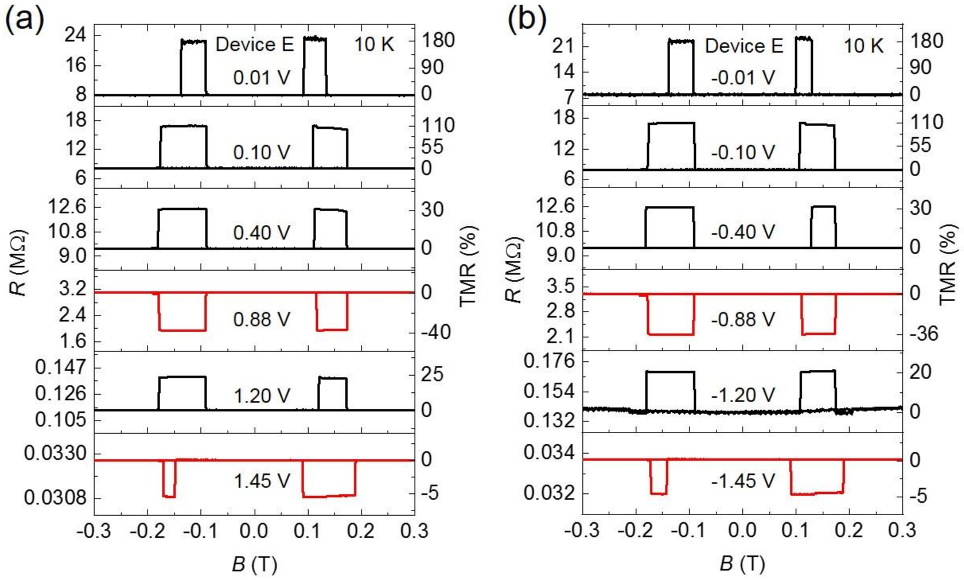

Figure S8. *R*-*B* curves and the corresponding TMR of device E with 9.2-nm-thick GaSe-layer at various positive bias (a) and negative bias (b). The temperature is fixed at 10 K.

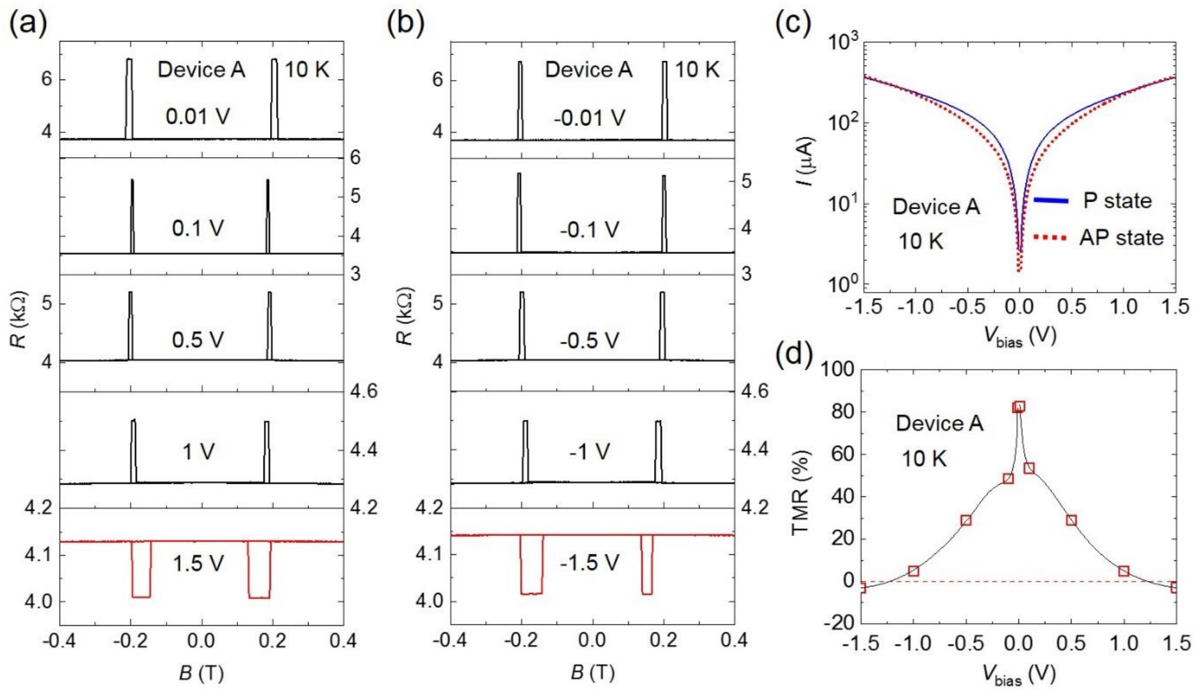

Figure S9. *R*-*B* curves of device A with 5.5-nm-thick GaSe-layer at various positive bias (a) and negative bias (b). (c) I-V_{bias} curves of device A measured in parallel and antiparallel magnetic configurations, respectively. (d) TMR of device A as a function of V_{bias}, which decreases with the increase of bias and becomes negative when the bias exceeds 1.20 V. The temperature is fixed at 10 K.

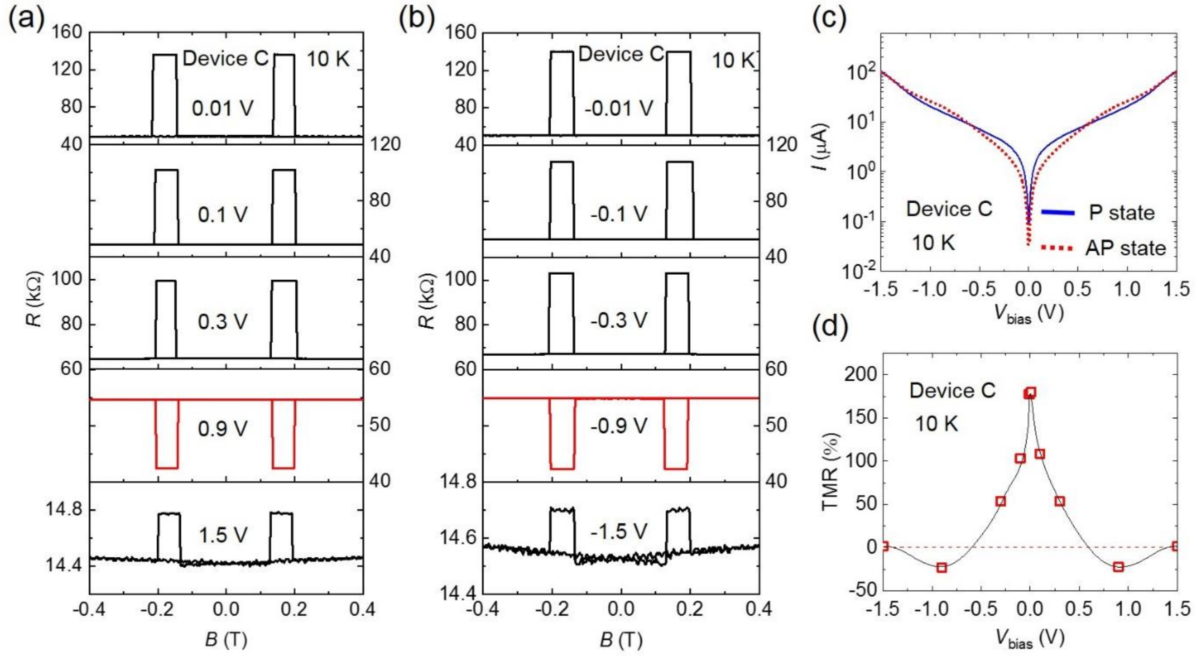

Figure S10. *R*-*B* curves of device C with 7.3-nm-thick GaSe-layer at various positive bias (a) and negative bias (b). (c) *I*-V_{bias} curves of device C measured in parallel and antiparallel magnetic configurations, respectively. (d) TMR of device C as a function of V_{bias}, which decreases with the increase of bias and becomes negative when the bias exceeds 0.60 V and then back to positive value when the bias exceeds 1.43 V. The temperature is fixed at 10 K.

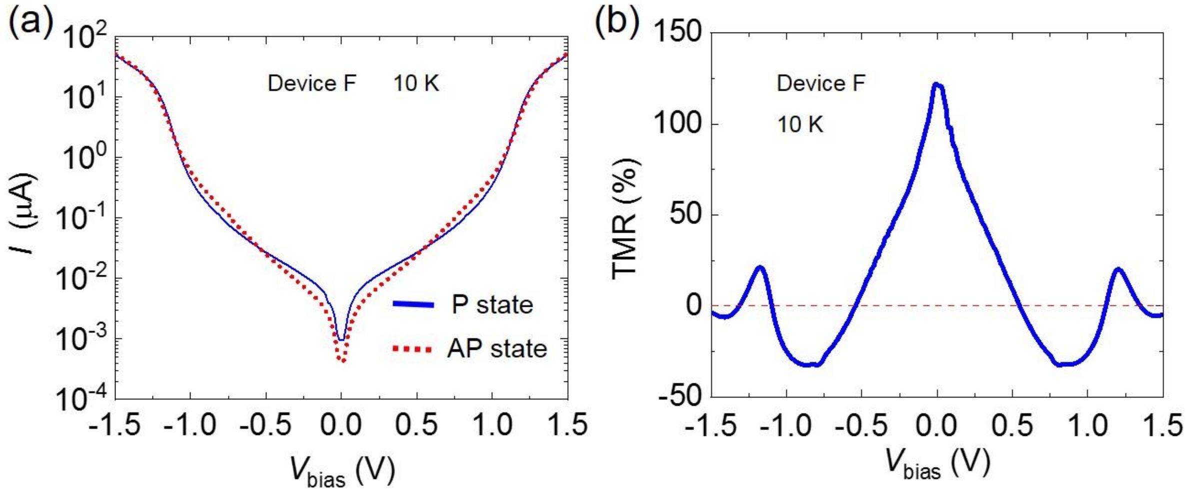

Figure S11. (a) *I*-V_{bias} curves of device F with 10-nm-thick GaSe-layer in parallel and antiparallel states, respectively. (b) TMR versus V_{bias} of device D. The temperature is $T = 10$ K.

Supplementary Note 7. The spin-resolved DOS simulated by DFT

Ab Initio Calculations: Our first-principles calculations are performed by the density functional theory (DFT) using the Vienna *ab initio* Simulation Package (VASP) code[14,15]. The exchange and correlation functionals are implemented by the generalized gradient approximation (GGA) of the Perdew-Burke-Ernzerhof functional[16]. A 2D FGT/GaSe/FGT van der Waals (vdW) heterostructure is constructed by sandwiching a 6-layer-GaSe between two 3-layer-FGTs. The lattice constant for pure FGT and GaSe is 3.99 and 3.81 Å, respectively. To obtain the correct spin polarization of FGT, the lattice constant of FGT/GaSe/FGT heterostructure is chosen as for the lattice of FGT, 3.99 Å, with a lattice mismatch of 4.7% compared to that of GaSe. Three different configurations between FGT and GaSe are considered: (i) Ga and Se atoms in GaSe-layer atop on Fe and Te atoms in FGT layer, respectively, (ii) Ga and Se atoms in GaSe-layer atop on Ge and Fe atoms in FGT layer, respectively, and (iii) Ga and Se atoms in GaSe-layer atop on Te and Ge atoms in FGT layer, respectively. The corresponding energy of these arrangements is -53.369, -53.440, and -53.448 eV, respectively. We find that the most stable configuration is (iii), as shown in **Figure S12**. The thickness of vacuum layer about 15 Å is set to avoid interactions between adjacent layers. Structures are fully relaxed until the force converged on each atom to less than 10^{-2} eV/Å. In all calculations, the plane-wave cutoff energy is set to 350 eV; Brillouin zone is sampled using Γ-centered 15×15×1 Monkhorst-pack *k* mesh[17]; and the vdW interaction are corrected by DFT-D3 Grimme method[18].

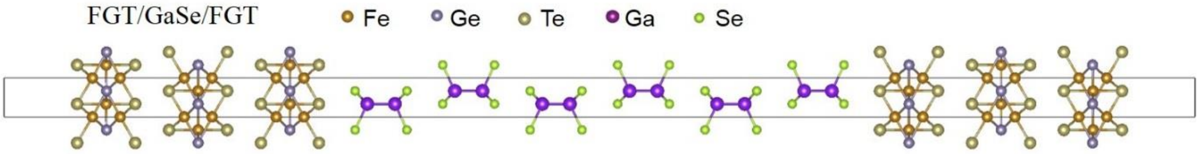

Figure S12. A side view of 3-layer-FGT/6-layer/3-layer-FGT atomic structure used for the calculations.

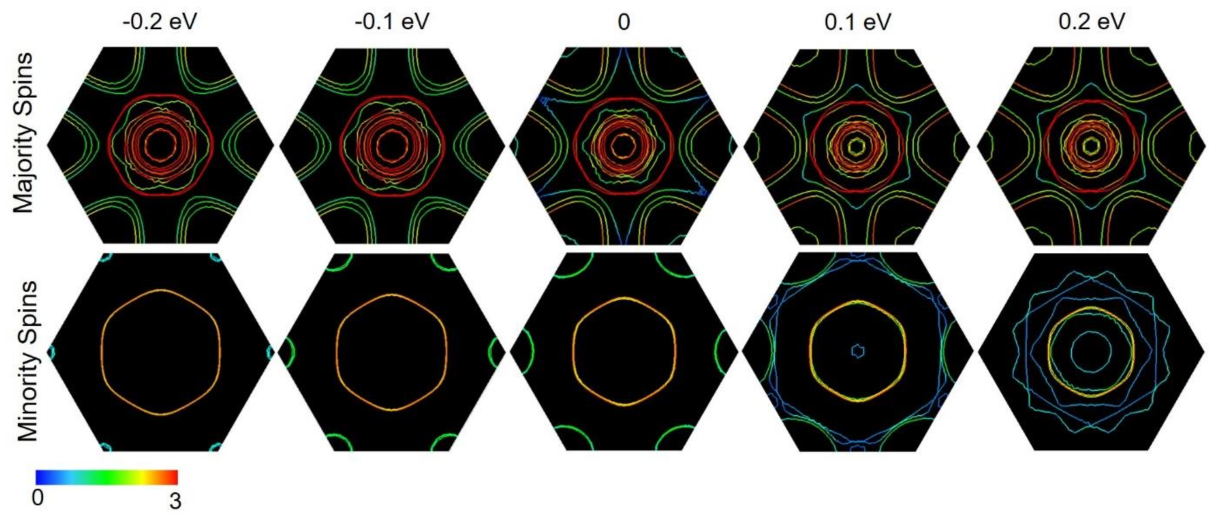

Figure S13. Majority and minority spins Fermi surfaces of three-layer Fe_3GeTe_2 at different energies ranging from E_F-0.2 to E_F+0.2 eV, where “0” indicates the Fermi energy. Colors indicate the Fermi surfaces belonging to different bands.

Figure S13 shows the majority and minority spins Fermi surfaces of three-layer Fe_3GeTe_2 at different energies ranging from E_F-0.2 to E_F+0.2 eV, where “0” indicates the Fermi energy. Majority spins have multiple bands at the Fermi energy covering the large portion of the 2D Brillouin zone (BZ), the minority spins have only a few states available, resulting in a large area of the 2D BZ with no overlap. Based on the Julliere model, this will give a large positive magnetoresistance. When voltage is applied to the MTJ device, the energy surface of one of the Fe_3GeTe_2 electrodes will move relatively to the other electrode, which makes the majority spins (or minority spins) of one electrode overlap with the minority spins (or majority spins) of the other electrode. As shown in **Figure S13**, in case of E_F+0.2 eV, some bands of majority and minority spins overlaps. As a result, the negative magnetoresistance will appear.

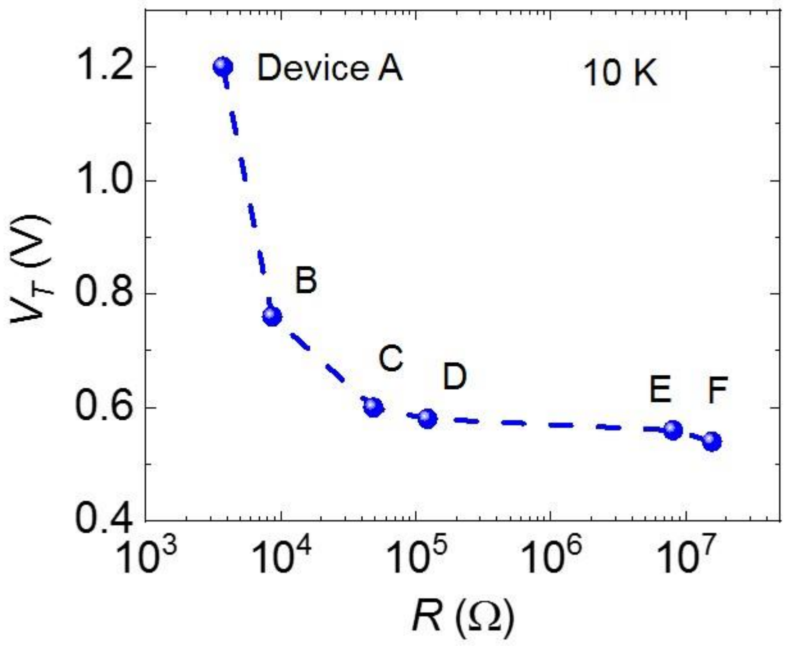

Figure S14. Bias voltage of the first TMR sign reversal at 10 K as a function of device resistance.

To investigate the relationship between thickness-dependent TMR sign reversal and device resistance, we extracted the bias voltage values of the first TMR sign reversal at 10 K as a function of device resistance, as shown in **Figure S14**. As the device resistance increases exponentially, the bias voltage values first rapidly decrease and then gradually stabilize. For devices A and B with lower resistance, the voltage dropped on tunnel junction is significantly smaller than the voltage applied, thus a larger bias voltage is required to reach the transition point of the first TMR sign reversal. With increasing the junction resistance, the voltage-divider effect becomes less important. This is why the difference of the voltage for the first TMR sign reversal between device D and E is very small.

Supplementary Note 8. The temperature-dependent TMR in the MTJ devices

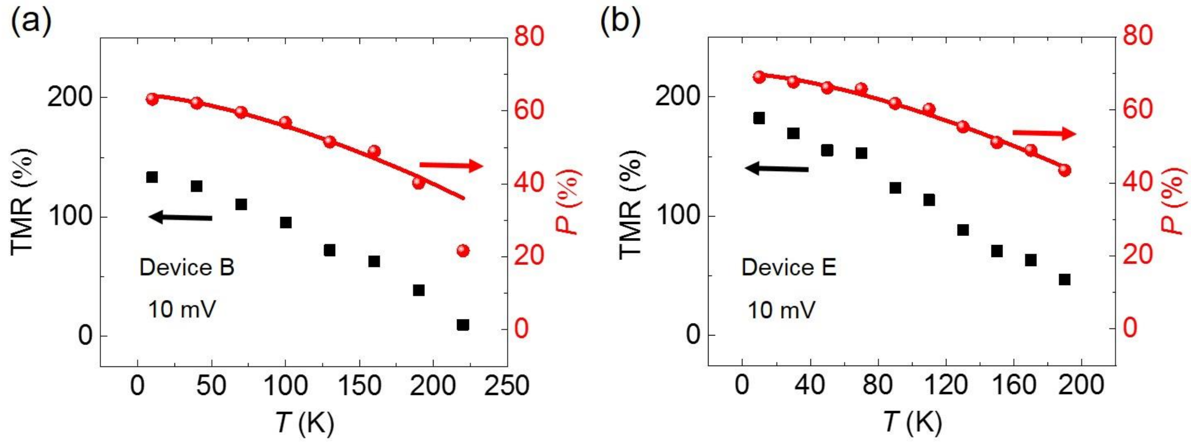

Figure S15. (a) The extracted TMR and the corresponding P of device B as a function of temperature at bias-voltage of 10 mV. The fitting value of α is 1.34×10^{-4} $K^{-3/2}$. (b) The extracted TMR and the corresponding P of device E as a function of temperature at bias-voltage of 10 mV. The fitting value of α is 1.40×10^{-4} $K^{-3/2}$.

References for Supplementary Information

1. Li, X. et al. Controlled Vapor Phase Growth of Single Crystalline, Two-Dimensional GaSe Crystals with High Photoresponse. *Sci. Rep.* **4**, 5497 (2014).
2. Cao, Y. et al. Strong enhancement of photoresponsivity with shrinking the electrodes spacing in few layer GaSe photodetectors. *Sci. Rep.* **5**, 8130 (2015).
3. Molas, M. R. et al. Raman spectroscopy of GaSe and InSe post-transition metal chalcogenides layers. *Faraday Discuss.* **227**, 163-170 (2021).
4. Hu, P. et al. Synthesis of Few-Layer GaSe Nanosheets for High Performance Photodetectors. *ACS Nano* **6**, 5988-5994 (2012).
5. Simmons, J. G. Generalized Formula for the Electric Tunnel Effect between Similar Electrodes Separated by a Thin Insulating Film. *J. Appl. Phys.* **34**, 1793-1803 (1963).
6. Le Toullec, R., Piccioli, N. & Chervin, J. C. Optical properties of the band-edge exciton in GaSe crystals at 10 K. *Phys. Rev. B* **22**, 6162 (1980).
7. Yıldırım, M. & Öksüzoğlu, R. M. Effect of bias voltage on tunneling mechanism in Co40Fe40B20/MgO/Co40Fe40B20 pseudo-spin valve. *J. Magn. Magn. Mater.* **379**, 280-287 (2015).
8. Chiu, F.-C. A Review on Conduction Mechanisms in Dielectric Films. *Adv. Mater. Sci. Eng.* **2014**, 578168 (2014).
9. Kim, S. et al. Highly Efficient Experimental Approach to Evaluate Metal to 2D Semiconductor Interfaces in Vertical Diodes with Asymmetric Metal Contacts. *ACS Appl. Mater. Interfaces* **13**, 27705-27712 (2021).
10. Morgan, C. et al. Impact of Tunnel-Barrier Strength on Magnetoresistance in Carbon Nanotubes. *Phys. Rev. Appl.* **5**, 054010 (2016).
11. Rashba, E. I. Theory of electrical spin injection: Tunnel contacts as a solution of the conductivity mismatch problem. *Phys. Rev. B* **62**, R16267(R) (2000).
12. Dolui, K., Narayan, A., Rungger, I. & Sanvito, S. Efficient spin injection and giant magnetoresistance in Fe/MoS_2/Fe junctions. *Phys. Rev. B* **90**, 041401(R) (2014).
13. Bing, D. et al. Optical contrast for identifying the thickness of two-dimensional materials. *Opt. Commun.* **406**, 128-138 (2018).
14. Kresse, G. & Hafner, J. Ab initio molecular-dynamics simulation of the liquid-metal--amorphous-semiconductor transition in germanium. *Phys. Rev. B* **49**, 14251 (1994).
15. Kresse, G. & Joubert, D. From ultrasoft pseudopotentials to the projector augmented-wave method. *Phys. Rev. B* **59**, 1758 (1999).
16. Perdew, J. P., Burke, K. & Ernzerhof, M. Generalized Gradient Approximation Made Simple. *Phys. Rev. Lett.* **77**, 3865 (1996).

17. Monkhorst, H. J. & Pack, J. D. Special points for Brillouin-zone integrations. *Phys. Rev. B* **13**, 5188 (1976).
18. Grimme, S., Antony, J., Ehrlich, S. & Krieg, H. A consistent and accurate ab initio parametrization of density functional dispersion correction (DFT-D) for the 94 elements H-Pu. *J. Chem. Phys.* **132**, 154104 (2010).
